# Antioxidant, antimicrobial and anticancer activity of the lichens *Cladonia furcata, Lecanora atra *and *Lecanora muralis*

**DOI:** 10.1186/1472-6882-11-97

**Published:** 2011-10-20

**Authors:** Branislav R Ranković, Marijana M Kosanić, Tatjana P Stanojković

**Affiliations:** 1 Department of Biology, Faculty of Science, University of Kragujevac, Radoja Domanovića 12, Kragujevac, Serbia; 2 Institute of Oncology and Radiology of Serbia, Pasterova 14, 11000 Belgrade, Serbia

**Keywords:** Lichens, Antioxidant activity, Antimicrobial activity, Anticancer activity

## Abstract

**Background:**

The aim of this study is to investigate in vitro antioxidant, antimicrobial and anticancer activity of the acetone extracts of the lichens *Cladonia furcata, Lecanora atra *and *Lecanora muralis*.

**Methods:**

Antioxidant activity was evaluated by five separate methods: free radical scavenging, superoxide anion radical scavenging, reducing power, determination of total phenolic compounds and determination of total flavonoid content. The antimicrobial activity was estimated by determination of the minimal inhibitory concentration by the broth microdilution method against six species of bacteria and ten species of fungi. Anticancer activity was tested against FemX (human melanoma) and LS174 (human colon carcinoma) cell lines using MTT method.

**Results:**

Of the lichens tested, *Lecanora atra *had largest free radical scavenging activity (94.7% inhibition), which was greater than the standard antioxidants. Moreover, the tested extracts had effective reducing power and superoxide anion radical scavenging. The strong relationships between total phenolic and flavonoid contents and the antioxidant effect of tested extracts were observed. Extract of *Cladonia furcata *was the most active antimicrobial agent with minimum inhibitory concentration values ranging from 0.78 to 25 mg/mL. All extracts were found to be strong anticancer activity toward both cell lines with IC_50 _values ranging from 8.51 to 40.22 μg/mL.

**Conclusions:**

The present study shows that tested lichen extracts demonstrated a strong antioxidant, antimicrobial and anticancer effects. That suggest that lichens may be used as as possible natural antioxidant, antimicrobial and anticancer agents to control various human, animal and plant diseases.

## Background

Continuous and uncontrolled use of synthetic drugs has led to the need to find new preparations of natural origin in the control and prevention of various human, animal and plant diseases. It is known that long-term use of synthetic drugs often causes numerous side effects and sometimes resistance [1]. Unlike synthetic drugs, bioactive natural products have beneficial effect on the whole organism and without causing unwanted effects. In search of new bioactive preparations of natural origin, lichens are the subject of many research teams.

Lichens are symbiotic organisms consisting of algae and fungi, and are important constituents of many ecosystems. They usually grow on rocks, non-fertile ground, as well as epiphytes on the trees and leaves [2]. These organisms are used for human nutrition, animal nutrition, for getting colours, perfumes and alcohol. Lichens have also, for hundreds of years, been used in many contry as a cure for diseases of humans. For example, *Lobaria pulmonaria *and *Parmelia sulcata *have been used in the treatment of pulmonary and cranial diseases, respectively. Similarly, *Xanthoria parietina *was used to cure jaundice and *Letharia vulpina *in stomach diseases [3-5]. The usage of some lichens for many years in the traditional medicine was later justified by numerous researches that confirmed their various biological activity.

Lichens produce secondary metabolites the "lichen substances", which comprise depsides, depsidones, dibenzofurans, xanthones and terpene derivatives. These metabolites sometimes make even more than 30% of the dry mass of talus. Lichens and their metabolites have manifold biological activity: antiviral, antibiotic, antitumor, allergenic, plant growth inhibitory, antiherbivore, ecological roles and enzyme inhibitory [3,6,7]. Because of that, the present study describes the evaluation of the antioxidant, antimicrobial and cytotoxic activities of the acetone extracts of the lichens *Cladonia furcata, Lecanora atra *and *Lecanora muralis*.

## Methods

### Lichen samples

Lichen samples of. *Cladonia furcata *(Hudson) Schrad.*, Lecanora atra *(Hudson) Ach. and *Lecanora muralis *(Schreber) Rabenh., were collected from Kopaonik, Serbia, in September of 2010. The demonstration samples are preserved in facilities of the Department of Biology and Ecology of Kragujevac, Faculty of Science. Determination of the investigated lichens was accomplished using standard methods.

### Preparation of the lichen extracts

Finely dry ground thalli of the investigated lichens (50 g) were extracted using acetone in a Soxchlet extractor. The extracts were filtered and then concentrated under reduced pressure in a rotary evaporator. The dry extracts were stored at -18°C until they were used in the tests [8]. The extracts were dissolved in 5% dimethyl sulphoxide (DMSO) for the experiments.

### Antioxidant activity

#### Scavenging DPPH radicals

The free radical scavenging activity of lichen extracts was measured by 1,1-diphenyl-2-picryl-hydrazil (DPPH). The method used is similar to the method previously used by some authors [9,10], but was modified in details. Two milliliters of methanol solution of DPPH radical in the concentration of 0.05 mg/mL and 1 mL of plant extract (1 mg/mL) were placed in cuvettes. The mixture was shaken vigorosly and alowed to stand at room temperature for 30 min. Then the absorbance was measured at 517 nm in spectrophotometer ("Jenway" UK). Ascorbic acid, butylated hydroxyanisole (BHA) and α-tocopherol were used as positive control. The DPPH radical concentration was calculated using the following equation:

DPPHscavengingeffect(%)=[(A0-A1)∕A0]×100,

where A0 is the absorbance of the negative control and A1 is the absorbance of reaction mixture or standards [11].

#### Reducing power

The reducing power of extracts was determined according to the method of Oyaizu [12]. One milliliter of extracts (1 mg/mL) were mixed with 2.5 mL of phosphate buffer (2.5 mL, 0.2 M, pH 6.6) and potassium ferricyanide [K_3_Fe(CN)_6_] (2.5 mL, 1%). The mixtures were incubated at 50°C for 20 min. Then, TCA (10%, 2.5 mL) was added to the mixture and centrifuged. Finally, the upper layer were mixed with distilled water (2.5 mL) and FeCl_3 _(0.5 mL; 0.1%). The absorbance of the solution was measured at 700 nm in spectrophotometer („Jenway" UK). Higher absorbance of the reaction mixture indicated that the reducing power is increased. Ascorbic acid, butylated hydroxyanisole (BHA) and α-tocopherol were used as positive control.

#### Superoxide anion radical scavenging activity

The superoxide anion radical scavenging activity of lichen extracts was detected according to the method of Nishimiki et al. [13]. Briefly, 0.1 mL of extracts (1 mg/mL) was mixed with 1 mL nitroblue tetrazolium (NBT) solution (156 μM in 0.1 M phosphate buffer, pH 7.4) and 1 mL NADH solution (468 μM in 0.1 M phosphate buffer, pH 7.4). The reaction was started by adding 100 μL of phenazine methosulphate (PMS) solution (60 μM in 0.1 M phosphate buffer, pH 7.4). The mixture was incubated at room temperature for 5 min, and the absorbance was measured at 560 nm in spectrophotometer („Jenway" UK) against blank samples. Decreased absorbance indicated increased superoxide anion radical scavenging activity. Ascorbic acid, butylated hydroxyanisole (BHA) and α-tocopherol were used as positive control. The percentage inhibition of superoxide anion generation was calculated using the following formula:

Superoxideanionscavengingactivity(%)=[(A0-A1)∕A0]×100,

where A0 is the absorbance of the negative control and A1 is the absorbance of reaction mixture or standards.

#### Determination of total phenolic compounds

Total soluble phenolic compounds in the lichen extracts were determined with Folin-Ciocalteu reagent according to the method of Slinkard and Singleton [14] using pyrocatechol as a standard phenolic compound. Briefly, 1 mL of the lichen extract (1 mg/mL) in a volumetric flasc diluted with distilled water (46 mL). One milliliter of Folin-Ciocalteu reagent was added and the content of the flask was mixed thoroughly. After 3 min 3 mL of Na_2_CO_3 _(2%) was added and then was allowed to stand for 2 h with intermittent shaking. The absorbance was measured at 760 nm in in spectrophotometer ("Jenway" UK). The total concentration of phenolic compounds in the extract detemined as microgram of pyrocatechol equivalent by using an equation that was obtained from standard pyrocatechol graph as

$$\begin{array}{c} \text{Absorbance}=0.0021\times \text{total}\;\text{phenols}\;\left[\mu \text{g}\;\text{pyrocatechol}\right]-0.0092\\ \left({\text{R}}^{2}=0.9934\right) \end{array}$$

#### Total flavonoid content

The total flavonoid content was determined using the Dowd method [15]. Two milliliters of 2% aluminium trichloride (AlCl_3_) in methanol was mixed with the same volume of the extract solution (1 mg/mL). The mixture was incubated at room temperature for 10 min, and the absorbance was measured at 415 nm in spectrophotometer („Jenway" UK) against blank samples. The total flavonoid content was detemined as microgram of rutin equivalent by using an equation that was obtained from standard rutin graph as

$$\begin{array}{c} \text{Absorbance}=0.0144\times \text{total}\;\text{flavonoid}\;\left[\mu \text{g}\;\text{rutin}\right]+0.0556\\ \left({\text{R}}^{2}=0.9992\right) \end{array}$$

### Antimicrobial activity

#### Microorganisms and media

The following bacteria were used as test organisms in this study: *Bacillus mycoides *(IPH 197), *Bacillus subtilis *(IPH 189), and *Staphylococcus aureus *(IPH 221) (Gram-positive bacteria); and *Enterobacter cloaceae *(IPH 241), *Escherichia coli *(IPH 246), and *Klebsiella pneumoniae *(IPH 251), (Gram-negative bacteria). All the bacteria used were isolates of the Institute for Protection of Health in Kragujevac (IPH) and the Faculty of Agriculture in Belgrade (FAB). Their identification was confirmed at the Microbiological Laboratory of Kragujevac, University of Kragujevac, Department of Biology. The fungi used as test organisms were: *Aspergillus flavus *(ATCC 9170), *Aspergillus fumigatus *(DBFS 310), *Botrytis cinerea *(DBFS 133), *Candida albicans *(IPH 1316), *Fusarium oxysporum *(DBFS 292), *Mucor mucedo *(ATCC 52568), *Paecilomyces variotii *(ATCC 22319), *Penicillium purpurescens *(DBFS 418), *Penicillium verrucosum *(DBFS 262), and *Trichoderma harsianum *(DBFS 379). They were from the mycological collection maintained by the Mycological Laboratory within the Department of Biology of Kragujevac University's Faculty of Science (DBFS). Bacterial cultures were maintained on Müller-Hinton agar substrates (Torlak, Belgrade). Fungal cultures were maintained on potato dextrose (PD) agar and Sabourad dextrose (SD) agar (Torlak, Belgrade). All cultures were stored at 4°C and subcultured every 15 days.

The sensitivity of microorganisms to acetone extracts of the investigated species of lichens was tested by determining the minimal inhibitory concentration (MIC).

Bacterial inoculi were obtained from bacterial cultures incubated for 24 h at 37°C on Müller-Hinton agar substrate and brought up by dilution according to the 0.5 McFarland standard to approximately 10^8 ^CFU/mL. Suspensions of fungal spores were prepared from fresh mature (3- to 7-day-old) cultures that grew at 30°C on a PD agar substrate. Spores were rinsed with sterile distilled water, used to determine turbidity spectrophotometrically at 530 nm, and then further diluted to approximately 10^6 ^CFU/mL according to the procedure recommended by NCCLS [16].

#### Minimal inhibitory concentration (MIC)

The minimal inhibitory concentration (MIC) was determined by the by the broth microdilution method with using 96-well micro-titer plates [17]. A series of dilutions with concentrations ranging from 50 to 0.195 mg/mL for extracts was used in the experiment against every microorganism tested. The starting solutions of extracts were obtained by measuring off a certain quantity of extract and dissolving it in DMSO. Two-fold dilutions of extracts were prepared in Müller-Hinton broth for bacterial cultures and SD broth for fungal cultures. The minimal inhibitory concentration was determined with resazurin. Resazurin is an oxidation-reduction indicator used for the evaluation of microbial growth. It is a blue non-fluorescent dye that becomes pink and fluorescent when reduced to resorufin by oxidoreductases within viable cells. The boundary dilution without any changing color of resazurin was defined as the minimal inhibitory concentration (MIC) for the tested microorganism at the given concentration. As a positive control of growth inhibition, streptomycin was used in the case of bacteria, ketoconazole in the case of fungi. A DMSO solution was used as a negative control for the influence of the solvents. All experiments were performed in triplicate.

### Cytotoxic activity

#### Cell lines

The human melanoma FemX and human colon carcinoma LS174 cell lines were obtained from the American Type Culture Collection (Manassas, VA, USA). Both cancer cell lines were maintained in the recommended RPMI-1640 medium supplemented with 10% heat-inactivated (56°C) fetal bovine serum, l-glutamine (3 mM), streptomycin (100 mg = mL), penicillin (100 IU = mL), and 25 mM HEPES and adjusted to pH 7.2 by bicarbonate solution. Cells were grown in a humidified atmosphere of 95% air and 5% CO_2 _at 37°C.

#### Treatment of cell lines

Stock solutions (100 mg/mL) of extracts, made in dimethylsulfoxide (DMSO), were dissolved in corresponding medium to the required working concentrations. Neoplastic FemX cells (5000 cells per well) and neoplastic LS174 cells (7000 cells per well) were seeded into 96-well microtiter plates, and 24 h later, after the cell adherence, five different, double diluted, concentrations of investigated compounds, were added to the wells. Final concentrations applied to target cells were 200, 100, 50, 25 and 12.5 μg/mL, except to the control wells, where only nutrient medium was added to the cells. Nutrient medium was RPMI 1640 medium, supplemented with l-glutamine (3 mM), streptomycin (100 lg/mL), and penicillin (100 IU/mL), 10% heat inactivated (56°C) fetal bovine serum (FBS) and 25 mM Hepes, and was adjusted to pH 7.2 by bicarbonate solution. The cultures were incubated for 72 hrs.

#### Determination of cell survival (MTT test)

The effect of extracts on cancer cell survival was determined by MTT test (microculture tetrazolium test), according to Mosmann [18] with modification by Ohno and Abe [19], 72 h upon addition of the compounds, as it was described earlier. Briefly, 20 μl of MTT solution (5 mg/mL PBS) were added to each well. Samples were incubated for further 4 h at 37 C in 5% CO2 and humidified air atmosphere. Then, 100 μl of 10% SDS were added to extract the insoluble product formazan, resulting from the conversion of the MTT dye by viable cells. The number of viable cells in each well was proportional to the intensity of the absorbance of light, which was then read in an ELISA plate reader at 570 nm. Absorbance (A) at 570 nm was measured 24 h later. To get cell survival (%), A of a sample with cells grown in the presence of various concentrations of the investigated extracts was divided with control optical density (the A of control cells grown only in nutrient medium), and multiplied by 100. It was implied that A of the blank was always subtracted from A of the corresponding sample with target cells. IC_50 _concentration was defined as the concentration of an agent inhibiting cell survival by 50%, compared with a vehicle-treated control. As a positive control was used *cis*-diamminedichloroplatinum (Cis-DDP). All experiments were done in triplicate.

#### Statistical analyses

Statistical analyses were performed with the EXCEL and SPSS softwares package. To determine the statistical significance of antioxidant activity, student's t-test were used. Pearson's bivariate correlation test was carried out to calculate correlation coefficients (r) between the content of total phenolic and flavonoid and the DPPH radical scavenging activity, reducing power and superoxide anion radical scavenging. All values are expressed as mean ± SD of three parallel measurements.

## Results

### Antioxidant activity

**Figure 1:**
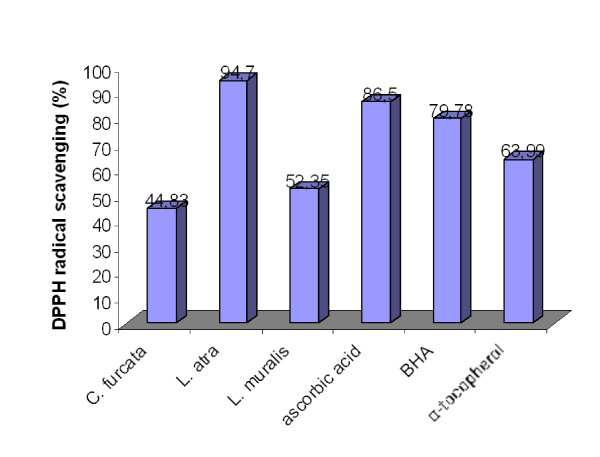
**DPPH radical scavenging of the acetone extracts of the lichens *Cladonia furcata, Lecanora atra *and *Lecanora muralis***.

The scavenging DPPH radicals of the studied lichen extracts is shown in Figure 1. Acetone extracts of the tested lichen showed a good scavenging activity on DPPH radical. There was a statistically significant difference between extracts and control (P < 0.05). The scavenging effects of all lichen extracts were 44.83 - 94.7%. Extracts from lichen *Lecanora atra *showed largest DPPH radical scavenging activity (94.7%) which was similar or greater than the standard antioxidants, ascorbic acid (86.5%), BHA (79.78%) and α-tocopherol (63.99%). The scavenging activity was also good for the lichen *Lecanora muralis *(52.35%). The lichen *Cladonia furcata *showed a slightly weaker DPPH radical scavenging activities (44.83%).

**Figure 2:**
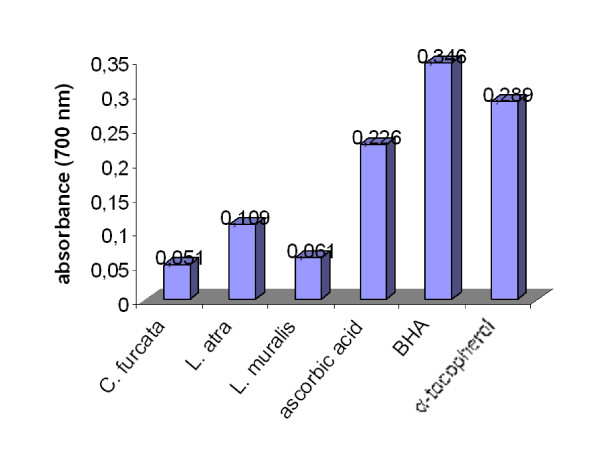
**Reducing power of the acetone extracts of the lichens *Cladonia furcata, Lecanora atra *and *Lecanora muralis***.

The results of the reducing power assay of lichen extracts are summarized in Figure 2. High absorbance indicates high reducing power. Measured values of absorbance varied from 0.051 to 0.109. Among the tested lichen species, *Lecanora atra *give highest reducing power. The reducing power in the acetone lichen extracts decreased in the following order: *Lecanora atra > Lecanora muralis > Cladonia furcata*.

**Figure 3:**
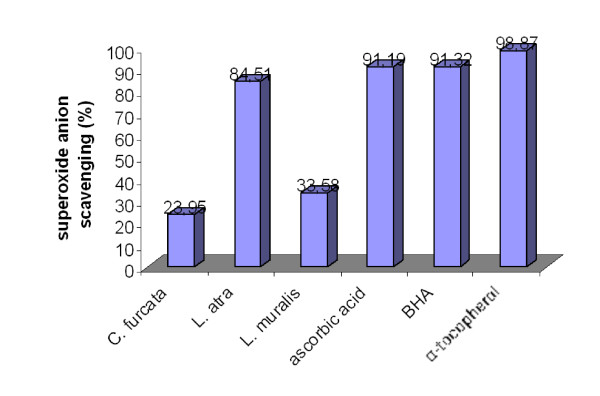
**Superoxide anion scavenging of the acetone extracts of the lichens *Cladonia furcata, Lecanora atra *and *Lecanora muralis***.

Results of superoxide anion scavenging activities of tested extracts are shown in Figure 3. All extracts revealed a relatively good superoxide anion scavenging activity. The superoxide anion scavenging activity for different lichens was within the range 23.95 - 84.51%. There was a statistically significant difference between extracts and control (P < 0.05). Maximum scavenging activity (84.51%) was in the acetone extracts of the lichen *Lecanora atra*. Acetone extract of lichen *Cladonia furcata *demonstrated weakest superoxide anion scavenging activity (23.95%).

Total phenolic and flavonoid constituents of tested extracts are given in Table 1. The amount of total phenolic compounds was determined as the pyrocatechol equivalent using an equation obtained from a standard pyrocatechol graph (y = 0.0021 × - 0.0092, R^2 ^= 0.9934). Highest phenolic compounds was identified in acetone extract of *Lecanora atra *at a 73.02 μg of pyrocatechol equivalent while acetone extracts of *Cladonia furcata *showed the lowest content at 12.95 μg of pyrocatechol equivalent. The amount of total flavonoid compounds was determined as the rutin equivalent using an equation obtained from a standard rutin graph (y = 0.0144 × + 0.0556, R^2 ^= 0.9992). The total flavonoid content for acetone extracts of *Cladonia furcata, Lecanora atra *and *Lecanora muralis *were 10.55, 54.77 and 34.56 μg of pyrocatechol equivalent, respectively.

**Table 1:** Total phenolics and flavonoid content of acetone extracts of *Cladonia furcata, Lecanora atra *and *Lecanora muralis*

Lichen species	Phenolics content μg of pyrocatechol equivalent	Flavonoid content μg of rutin equivalent
*C. furcata*	12.95 ± 1.065	10.55 ± 1.099
*L. atra*	73.02 ± 1.275	54.77 ± 1.231
*L. muralis*	43.19 ± 1.085	34.56 ± 1.074

The tested extracts exhibited the highest radical scavenging activity with the greatest amount of phenolic and flavonoid contents. Correlation coefficient between phenolic and flavonoid compounds of the tested extracts and free radical scavenging activity were r = 0.921 and r = 0.907, respectively.

Various antioxidant activities (DPPH radical scavenging, superoxide anion radical scavenging and reducing power) were compared to standard antioxidants such as ascorbic acid, butylated hydroxyanisole (BHA) and α-tocopherol. The results showed that standard antioxidants had similar or slightly stronger activity than tested extracts.

### Antimicrobial activity

The antimicrobal activity of the tested lichen extracts against the tested microorganisms was shown in the Table 2.

**Table 2:** Minimum inhibitory concentration (MIC) of acetone extracts of *Cladonia furcata, Lecanora atra *and *Lecanora muralis*

Lichen species	*C. furcata*	*L. atra*	*L. muralis*	S - K
*B. mycoides*	0.78^a^	1.56	3.12	7.81 -
*B. subtilis*	0.78	1.56	6.25	7.81 -
*E. cloacae*	0.78	3.12	3.12	1.95 -
*E. coli*	1.56	-	-	31.25 -
*K. pneumoniae*	0.78	3.12	3.12	1.95 -
*S. aureus*	0.78	3.12	3.12	31.25 -
*A. flavus*	25	25	-	- 3.9
*A. fumigatus*	12.5	25	-	- 3.9
*B. cinerea*	25	25	25	- 1.95
*C. albicans*	6.25	12.5	-	- 1.95
*F. oxysporum*	25	25	-	- 3.9
*M. mucedo*	25	25	-	- 31.25
*P. variotii*	12.5	25	25	- 1.95
*P. purpurescens*	25	25	-	- 3.9
*P. verrucosum*	25	25	-	- 7.81

^a^Minimum inhibitory concentration (MIC); values given as mg/ml for lichen extract and as μg/ml for antibiotics Values are the mean of three replicate.

Antibiotics: K - ketoconazole, S - streptomycin.

The maximum antimicrobial activity was found in the acetone extract of the lichen *Cladonia furcata*, which, in relatively low concentrations inhibited the tested bacteria and fungi. This lichen inhibited all of the tested bacteria in concetracion of 0.78 mg/mL, except *Escherichia coli *(MIC = 1.56 mg/mL). MIC for fungi ranged from 6.25 to 25 mg/mL.

Extract of the lichen *Lecanora atra *manifested relatively strong antimicrobial activity. This lichen inhibited all of the tested bacteria, except *Escherichia coli*, which was resistant. The MIC for bacteria ranged from 1.56 mg/mL against *Bacillus mycoides *and *Bacillus subtilis *to 3.12 mg/mL against *Enterobacter cloacae, Klebsiella pneumoniae *and *Staphylococcus aureus*. MIC for fungi ranged from 12.5 against *Candida albicans *to 25 mg/mL against other fungi.

The lichen *Lecanora muralis *manifested lowest antimicrobial activity. The MIC for bacteria ranged from 3.12 to 6.25 mg/mL (except *Escherichia coli*, which was resistant). Antifungal activity was found only against *Botrytis cinerea *and *Paecilomyces variotii*. Measured MIC value for both fungi was 25 mg/mL.

The antimicrobial activity was compared with the standard antibiotics, streptomycin (for bacteria) and ketoconazole (for fungi). The results showed that standard antibiotics had stronger activity than tested extracts as shown in Table 2. In a negative control, DMSO had no inhibitory effect on the tested organisms.

### Cytotoxic activity

The cytotoxic activity of the studied lichen extracts related to tested cell lines was shown in the Table 3.

**Table 3:** Growth inhibitory effects of acetone extracts of *Cladonia furcata, Lecanora atra *and *Lecanora muralis *on FemX and LS 174 cell lines

	FemX	LS 174
**Lichen species**	**IC_50 _(μg/ml)**	

*C. furcata*	23.52 ± 1.08	40.22 ± 0.03
*L. atra*	8.51 ± 0.05	10.29 ± 0.51
*L. muralis*	9.58 ± 0.38	12.23 ± 0.95
Cis-DDP	0.94 ± 0.35	2.3 ± 0.31

The tested lichen extracts manifested a strong cytotoxic activity against target cells *in vitro*. The inhibition concentration at 50% inhibition (IC_50_) was the parameter used to compare the cytotoxic activity. A lower IC_50 _meant better cytotoxic activity.

The *Lecanora atra *was exhibited the best cytotoxic activity. The IC_50 _against FemX and LS174 cell lines was 8.51 and 10.29 μg/mL, respectively.

The extract of *Lecanora muralis *also showed a good cytotoxic activity against both cell lines. The IC_50 _value was 9.58 μg/mL against FemX cell and 12.23 μg/mL against LS174 cell.

The *Cladonia furcata *manifested a slightly weaker cytotoxic activity. The IC_50 _value was 23.52 μg/mL related to FemX cell and 40.22 μg/mL related to LS174 cell.

As shown in table, pozitive control (Cis-DDP) had slightly better cytotoxic activity than tested lichen extracts.

## Discusion

In the present study, *in vitro *antioxidant, antimicrobial and cytotoxic activities of acetone extract from the lichens *Cladonia furcata, Lecanora atra *and *Lecanora muralis *were examined.

The tested lichen extracts have a strong antioxidant activity against various oxidative systems *in vitro*. We found that the tested extracts exhibited the highest radical scavenging activity with the greatest amount of phenolic content. The highest value of phenols was seen in the acetone extract of *Lecanora atra *which exhibited the strongest radical scavenging activity. Based on these results, it could be concluded that antioxidative nature of the extracts might depend on their phenolics. Phenolic components are potential antioxidants, free radical terminators [20,21]. These compounds are the main agents that can donate hydrogen to free radicals and thus break the chain reaction of lipid oxidation at the first initiation step. This high potential of phenolic compounds to scavenge radicals such as singlet oxygen, superoxide and hydroxyl radicals may be explained by their phenolic hydroxyl groups [22]. Flavonoids are also the most important natural phenolics and they possess a broad spectrum of chemical and biological activities including radical scavenging properties [23]. Numerous researches found a high correlations between antioxidative activities and phenolic content [24-26]. Interestingly, Odabasoglu et al. [25] reported that in some lichens extracts no correlation was found between the total phenol and the antioxidant activity, suggesting that the antioxidant activity of different lichens may also depend on other, non-phenol components. Antioxidant effect of some other lichens was also studied by other researchers. For example, Gulcin et al. [27] found that the aqueous extracts of *Cetraria islandica *had a strong antioxidant activity. Similar results were reported by Behera et al. [28] for different extracts from the lichen *Usnea ghattensis*. Kekuda et al. [29] find an antioxidant activity for the extracts of the lichen *Parmotrema pseudotinctorum *and *Ramalina hossei*. Manojlović et al. [30] explored antioxidant properties of *Laurera benguelensis*.

In our experiments, the tested lichen extracts show a relatively strong antimicrobial activity. The intensity of the antimicrobial effect depended on the species of lichen, its concentration and the tested organism. The extract of *Cladonia furcata *had the strongest antimicrobial activity among the tested species in this study, inhibiting the tested bacteria and fungi at low concentrations, while the lowest activity showed *Lecanora muralis*. Differences in antimicrobial activity of different species of lichens are probably a consequence of the presence of different components with antimicrobial activity [31,32,24].

The extracts used in this study, had a stronger antibacterial than antifungal activity. This observation is in accordance with other studies [33,24], focused on the antimicrobial activity which have demonstrated that bacteria are more sensitive to the antimicrobial activity than the fungi due to differences in the composition and permeability of the cell wall. The cell wall of Gram-positive bacteria is made of peptidoglucanes and teichoic acids, while the cell wall of Gram-negative bacteria is made of peptidoglucanes, lipopolysacharides and lipoproteins [34,35]. The cell wall of fungi is poorly permeable and it consists of polysaccharides such as hitchin and glucan [36].

Numerous lichens were screened for antimicrobial activity in search of the new antimicrobial agents. Ranković et al. [24] find an antimicrobial activity for the methanol extract of the lichens *Parmelia centrifuga*. Similar results were reported by Candan et al. [37] for different extracts extracted from the lichen *Parmelia sulcata*. Goel et al. [38] found out that lichen *Parmelia reticulata *had a strong antimicrobial influence.

In present study, the results clearly demonstrate that acetone extracts of studied lichens induced significant cytotoxic effect on the tested cancer cell lines. Until now, only few research proved that lichen extracts have anticancer activity. Bezivin et al. [39] reported significant anticancer effect for *Parmelia caperata, Cladonia convoluta, Cladonia rangiformis, Platisma glauca *and *Ramalina cuspidata*. Manojlović et al. [40] explored anticancer properties of *Thamnolia vermicularis*. Trigiani et al. [41] found strong anticancer activity for *Xanthoria parietina*.

Some literature data reported that lichen components are responsible for anticancer activities of lichens. Anticancer activity of various lichens components are known, such as: usnic acid, lecanoric acid, gyrophoric acid, salazinic acid, lobaric acid, evernic acid, vulpinic acid, protolichesterinic acid [42,43]. However, it is difficult to determine the contribution of individual components for the overall anticancer effect. Often, the activity of the extracts may be the result of an synergistic effect of several compounds.

## Conclusion

It conclusion, it can be stated that tested lichen extracts have a strong antioxidant, antimicrobial and anticancer activity *in vitro*. On the basis of these results, lichen appear to be good and safe natural antioxidant, antimicrobial and anticancer agents and also, could be of significance in human therapy, animal and plant diseases. Further studies should be done to search new compounds from lichens that exhibit strong antioxidant, antimicrobial and anticancer activity.

## Competing interests

The authors declare that they have no competing interests.

## Authors' contributions

MMK and TPS conducted experiments on antioxidant activity, antimicrobial activity and anticancer activity of tested lichens. BRR participated in design of the study and preparation of the manuscript. All the authors read and approved the final manuscript.

## Pre-publication history

The pre-publication history for this paper can be accessed here:

http://www.biomedcentral.com/1472-6882/11/97/prepub
